# Editorial: Macrophages: allies or merciless enemies in musculoskeletal systems

**DOI:** 10.3389/fimmu.2025.1746089

**Published:** 2025-12-16

**Authors:** M. Alaa Terkawi, Ken Kadoya, Tsutomu Endo

**Affiliations:** Department of Orthopedic Surgery, Faculty of Medicine and Graduate School of Medicine, Hokkaido University, Sapporo, Japan

**Keywords:** musculoskeletal disorders, macrophage, homeostasis, pathogenesis, therapies

Musculoskeletal disorders, including osteoarthritis (OA), rheumatoid arthritis (RA) and osteoporosis (OP) impose a substantial global economic and social burden, significantly reducing individuals’ mobility and overall quality of life, and consequently diminishing their productivity in society. Current therapeutic agents for these diseases often fail to fully restore the structure and function of affected tissues, highlighting the need for innovative treatment strategies (1). One promising approach involves mimicking the intrinsic cellular mechanisms that maintain and restore tissue homeostasis. Achieving this, however, requires a deeper understanding of the cellular networks and molecular signaling pathways that regulate these processes within musculoskeletal tissues.

Among the many cell types that shape musculoskeletal tissue health, macrophages stand out as central players in maintaining balance, coordinating immune defense, and guiding tissue repair. These highly versatile immune cells exhibit remarkable plasticity, enabling them to dynamically adjust their phenotypic and functional states in response to local microenvironmental cues. Macrophages can adopt a wide spectrum of activation states that reflect the specific physiological or pathological conditions of their resident tissues. Traditionally, activated macrophages have been classified into two major phenotypes, namely the classically activated, pro-inflammatory M1 type and the alternatively activated, anti-inflammatory M2 type. However, recent single-cell RNA sequencing studies have revealed a far more complex landscape, showing that macrophage phenotypes exist along a continuum shaped by tissue-specific and environmental factors. Transcriptomic analyses of macrophages derived from OA, RA, and OP tissues confirm that the traditional M1/M2 model inadequately captures their diversity (2, 3). Understanding the context-dependent activation states and molecular mechanisms that govern macrophage polarization is therefore critical for developing next-generation therapeutic strategies. In response to these scientific challenges, this Research Topic brings together four review articles and one original research article that collectively explore the multifaceted roles of macrophages in homeostatic, reparative and pathological processes within musculoskeletal tissues.

The significance of this research field is underscored in the review, “Unveiling Research Hotspots: A Bibliometric Study on Macrophages in Musculoskeletal Diseases.” In this work, Yue et al. perform an automated bibliometric analysis to map research trends and key focus areas related to macrophages in musculoskeletal disorders. The study provides a comprehensive overview of the field’s evolution, demonstrating that research on macrophages in musculoskeletal diseases has expanded rapidly over the past two decades. Data were extracted from articles published between 2004 and 2023, and the analysis highlights emerging topics that may guide future investigations into macrophage-mediated mechanisms in musculoskeletal health and disease.

Functionally, macrophages act as sentinels of the immune system, serving as the first line of defense against invading pathogens through their ability to eliminate them by phagocytosis. Beyond their antimicrobial role, they are crucial for maintaining tissue homeostasis by preventing the accumulation of harmful substances through efferocytosis, the process of clearing apoptotic cells and debris, and by promoting the resolution of inflammation. Efferocytic macrophages play an essential role in the resolution of inflammation and initiating reparative processes in musculoskeletal tissues (4). For instance, effective efferocytosis by osteal macrophages is indispensable for skeletal homeostasis as the failure of this process leads to the accumulation of apoptotic osteoblasts and residual bone materials at resorptive sites, thereby triggering inflammatory bone loss (5). Another homeostatic function of macrophages is their involvement in systemic metabolic regulation, where they influence lipid turnover and iron homeostasis, both of which are essential for skeletal muscle regeneration and bone repair and remodeling (6, 7). Moreover, macrophages play central roles in regulating inflammation, promoting tissue repair, and coordinating remodeling processes through the secretion of cytokines, growth factors, and matrix-modifying enzymes that drive angiogenesis and extracellular matrix reconstruction. In the review “Macrophages and Osteoclasts: Similarity and Divergence Between Bone Phagocytes” by Halper et al. the authors explore the shared progenitors, immune functions, and roles in tissue remodeling of osteoclasts and macrophages. Both cell types exhibit the phenotypic and functional plasticity characteristic of the myeloid lineage, which is shaped by their origin and microenvironment. Within the bone milieu, macrophages and osteoclasts are closely related immune cells, as macrophages primarily function in pathogen clearance and tissue repair, while osteoclasts are specialized in bone resorption. Halper et al. propose that identifying precise molecular targets to modulate macrophage and osteoclast activity could yield more selective and effective treatments with fewer side effects.

In addition to their homeostatic and reparative roles in immune and tissue regulation, proinflammatory macrophages, traditionally referred to as M1 macrophages, play a pivotal role in the pathogenesis of musculoskeletal diseases by initiating acute inflammatory responses and promoting tissue remodeling/damage. Likewise, senescent macrophages have been shown to contribute to chronic inflammation, driving cartilage degradation and aberrant bone remodeling within the joint and bone microenvironment, particularly in OA and OP (3). Conversely, excessive activation of anti-inflammatory macrophages, traditionally referred to as M2 macrophages, can lead to pathological fibrosis, as they secrete mediators that promote synovial fibrosis, joint degeneration, and muscle damage (8). In “The Multifaceted Role of Macrophages in Homeostatic and Injured Skeletal Muscle,” the authors highlight the complex involvement of macrophages in skeletal muscle physiology. Under normal conditions, macrophages clear necrotic debris and secrete trophic factors that promote myofiber regeneration, but during chronic injury, muscular dystrophy, or aging, their aberrant activation can impair tissue repair. Aging further alters macrophage heterogeneity and function, exacerbating sarcopenia and delayed healing. This review underscores the importance of macrophage balance for maintaining muscle integrity throughout life. Complementing this, Zhu et al., in “Potential Therapeutic Targets of Macrophages in Inhibiting Immune Damage and Fibrotic Processes in Musculoskeletal Diseases,” identify key factors regulating macrophage activity in muscle pathology, highlighting macrophage-driven fibrosis as a central feature, and underscore the potential of macrophage-targeted therapies, such as adoptive transfer of polarized macrophages and miRNA-based epigenetic interventions, which hold promise for clinical translation in musculoskeletal disorders. In the research article “FBXL3 Serves as a Suppressor of Regenerative Myogenesis” by Wang and Zhou, FBXL3 is identified as a negative regulator of skeletal muscle differentiation, with its deletion in satellite cells shown to enhance myogenic regeneration by promoting the proteasomal degradation of TCF12. Although this study does not directly examine macrophage function, the FBXL3-associated signaling pathway may intersect with macrophage-mediated inflammatory and regenerative processes in skeletal muscle.

Collectively, these studies underscore the pivotal role of macrophages as regulators of tissue homeostasis, inflammation, and repair across the musculoskeletal system. The heterogeneous nature of tissue macrophages, supported by recent single-cell RNA sequencing data showing phenotypes exist along a spectrum shaped by tissue and environmental factors, underscores the need to move beyond the simple M1/M2 classification, with a new function-based framework offering a potentially more meaningful approach. A deeper understanding of the molecular and cellular pathways governing macrophage behavior provides a foundation for macrophage-targeted therapies designed not only to resolve inflammation but also to promote genuine tissue regeneration and restore homeostatic balance.
